# MOF-Derived Co and Fe Species Loaded on N-Doped Carbon Networks as Efficient Oxygen Electrocatalysts for Zn-Air Batteries

**DOI:** 10.1007/s40820-022-00890-w

**Published:** 2022-08-11

**Authors:** Yuanyuan Xue, Yibo Guo, Qinming Zhang, Zhaojun Xie, Jinping Wei, Zhen Zhou

**Affiliations:** 1 School of Materials Science and Engineering, Institute of New Energy Material Chemistry, Key Laboratory of Advanced Energy Materials Chemistry (Ministry of Education), Renewable Energy Conversion and Storage Center (ReCast), Nankai University, Tianjin, 300350 People’s Republic of China; 2 School of Chemical Engineering, Zhengzhou University, Zhengzhou, 450001 People’s Republic of China

**Keywords:** Oxygen reduction reaction, Oxygen evolution reaction, Zn-air batteries, Bifunctional catalysts, Metal-organic frameworks

## Abstract

**Highlights:**

- A novel method is developed to prepare bifunctional oxygen electrocatalysts composed of Co nanoparticles and highly dispersed Fe loaded on N-doped carbon substrates by virtues of metal-organic frameworks and two different doping processes.
- The designed catalysts show comparable performance with commercial catalysts. Meanwhile, rechargeable Zn-air batteries with prepared catalysts demonstrate high peak power density and good cycling stability.
- The performance promotion originates from the synergy between Co nanoparticles and highly dispersed Fe, porous structures, large specific areas, and distinct three-dimensional carbon networks.

**Abstract:**

Searching for cheap, efficient, and stable oxygen electrocatalysts is vital to promote the practical performance of Zn-air batteries with high theoretic energy density. Herein, a series of Co nanoparticles and highly dispersed Fe loaded on N-doped porous carbon substrates are prepared through a “double-solvent” method with in situ doped metal-organic frameworks as precursors. The optimized catalysts exhibit excellent performance for oxygen reduction and evolution reaction. Furthermore, rechargeable Zn-air batteries with designed catalysts demonstrate higher peak power density and better cycling stability than those with commercial Pt/C+RuO_2_. According to structure characterizations and electrochemical tests, the interaction of Co nanoparticles and highly dispersed Fe contributes to the superior performance for oxygen electrocatalysis. In addition, large specific surface areas, porous structures and interconnected three-dimensional carbon networks also play important roles in improving oxygen electrocatalysis. This work provides inspiration for rational design of advanced oxygen electrocatalysts and paves a way for the practical application of rechargeable Zn-air batteries.
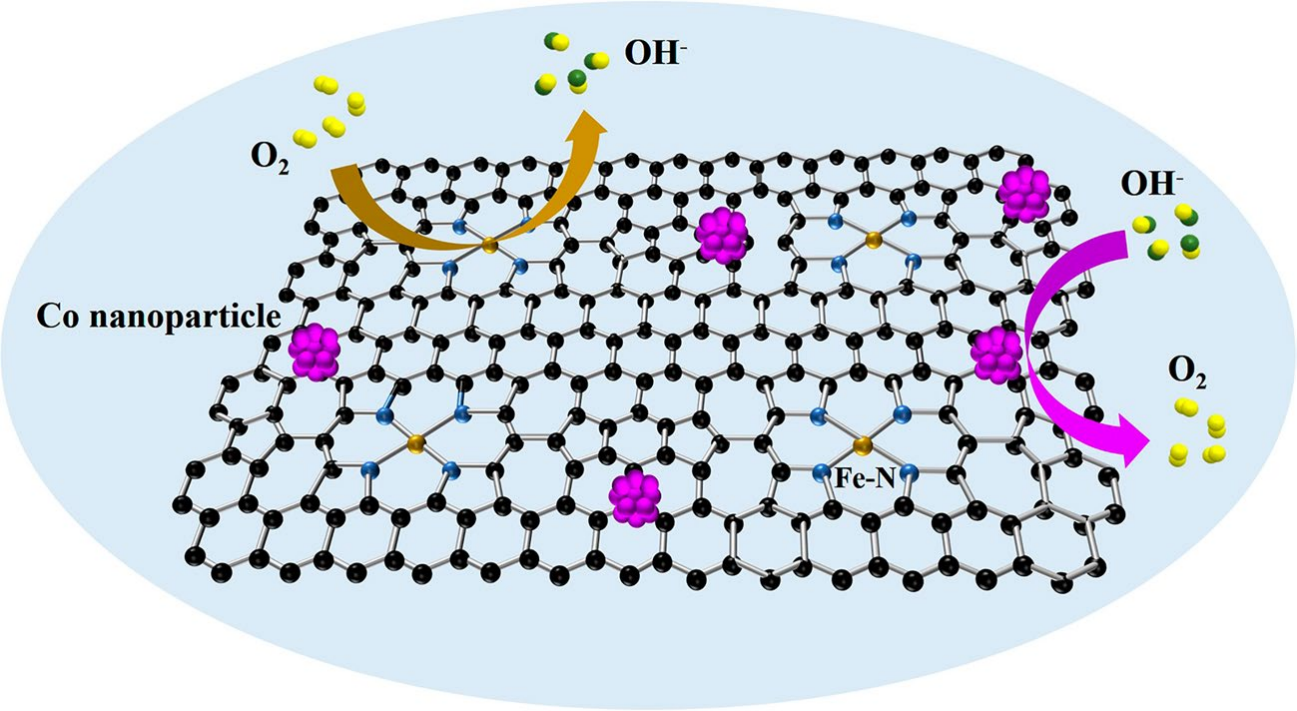

**Supplementary Information:**

The online version contains supplementary material available at 10.1007/s40820-022-00890-w.

## Introduction

For the demand of carbon neutrality, developing sustainable energy storage and conversion technologies is essential [1–5]. Rechargeable Zn-air batteries have attracted much interest due to their ultrahigh theoretical energy density, safety and low cost [6–8]. However, the practical application of rechargeable Zn-air batteries is seriously hindered by the sluggish kinetics of oxygen cathodes [9]. For rechargeable Zn-air batteries, oxygen reduction reaction (ORR) and oxygen evolution reaction (OER) are conducted at oxygen cathodes during discharging and charging processes, respectively [10]. Noble metal-based catalysts, such as Pt-based and IrO_2_-based catalysts, are efficient catalysts for speeding ORR or OER processes. However, the scarcity, high cost, single functionality and instability of these noble metal catalysts hinder the practical application. For searching for cheap, efficient, bifunctional and durable alternatives of noble metal catalysts [2, 11–13], transition metal-based materials loaded on carbon substrates demonstrate great potential for ORR and OER [14–19], since carbon substrates have high electrical conductivity and transition metals could serve as active sites for ORR/OER. However, rationally constructing bifunctional transition metal/carbon composite catalysts remains a challenge [20–22]. There are two reasons: First, it is still difficult to accomplish the tight connection between transition metals and carbon substrates and the uniform dispersion of one or more transition metals on carbon substrates simultaneously; Second, the structure-activity relationship of transition metal/carbon composites is obscure.

Metal-organic frameworks (MOFs), in which transition metal ions as nodes connect with organic ligands to form periodic porous structures, possess controllable pores, large specific surface areas, and tunable structures and compositions [23]. They are one class of promising materials in catalysis, energy storage, gas adsorption/separation, etc. [24–26]. Metal elements originate from nodes of MOFs and doping processes could uniformly disperse on carbon skeletons by the calcination of MOF precursors [18, 27, 28]. Therefore, with the assistance of MOFs as precursors, transition metal/carbon composite catalysts with tight connection and uniform distribution could be facilely prepared [29–31]. Meanwhile, MOF-derived metal/carbon composite catalysts could inherent the morphology and porous structure of MOFs, which are beneficial for the exposure of active sites, electron transfer and mass transportation in electrocatalytic processes [27].

ORR and OER go through distinct reaction pathways, and thus require different active centers [32]. Therefore, it is necessary to design catalysts with diverse active sites to realize bifunctional oxygen electrocatalysis. Fe loaded on N-doped carbon materials (FeNC) have been recognized as highly active sites for ORR [33–37], while Co nanoparticles interacted with carbon substrates could improve OER performance [38, 39]. Thus, it is beneficial for the accomplishment of bifunctional oxygen electrocatalysis, by constructing a single catalyst composed of Fe species and Co nanoparticles supported on carbon substrates. Bearing these in mind, we developed a novel method to prepare Co nanoparticles and highly dispersed Fe loaded on N-doped porous carbon substrates (CoNP@FeNC) for oxygen electrocatalysis. The confinement growth of metal nanoparticles and the high dispersion of metal atoms were accomplished simultaneously, by the assistance of the novel method. CoNP@FeNC catalysts were prepared by ZIF-8 (zeolitic imidazolate framework-8) as the host and two doping methods to incorporate Fe and Co elements. The morphology and structure of CoNP@FeNC catalysts were precisely characterized and the performances for ORR and OER were systematically investigated.

## Experimental Section

### Materials Preparation

#### Preparation of FeNC

Solution *A* was achieved by dissolving 1.97 g 2-methylimidazole in 150 mL methanol, and 1.695 g Zn(NO_3_)_2_·6H_2_O and 60 mg Fe(NO_3_)_3_·9H_2_O were dissolved in 150 mL methanol to form Solution *B*. Then Solutions *A* and *B* were mixed. The mixture was kept static at 60 °C for 12 h, and stirred for another 12 h. The obtained precipitates were centrifuged and washed with methanol for several times and dried in vacuum at 60 °C to obtain Fe-doped ZIF-8. Finally, Fe-doped ZIF-8 was placed in a tube furnace and heated to 1000 °C for 1 h in a stream of Ar to yield FeNC.

#### Preparation of CoNP@NC

Similarly, 1.97 g 2-methylimidazole was dissolved in 150 mL methanol to form Solution A, and 1.695 g Zn(NO_3_)_2_·6H_2_O was dissolved in 150 mL methanol to form Solution *B*. Then Solutions *A* and *B* were mixed. The mixture was stirred for 12 h. The obtained precipitates were centrifuged and washed with methanol for several times and dried in vacuum at 60 °C to obtain ZIF-8. 200 mg ZIF-8 were dissolved in 24 mL n-hexane under ultrasound for 1 h at room temperature to form Suspension *C*. Solution *D* was prepared by dissolving 29 mg Co(NO_3_)_2_·6H_2_O in 280 μL methanol, and was dropwise added to Suspension *C* under ultrasound. Then the mixture was stirred for 2 h. The obtained precipitates were centrifuged and washed with methanol for several times and dried in vacuum at 60 °C to obtain Co-doped ZIF-8. Finally, Co-doped ZIF-8 was placed in a tube furnace and heated to 1000 °C for 1 h in a stream of Ar to yield CoNP@NC.

#### Preparation of CoNP@FeNC

200 mg Fe-doped ZIF-8 were dissolved in 24 mL n-hexane under ultrasound for 1 h at room temperature to form Suspension *A*, and 29 mg Co(NO_3_)_2_·6H_2_O (0.05 mmol) was dissolved in 280 μL methanol to form Solution *B*. Solution *B* was dropwise added to Suspension *A* under ultrasound. Then the mixture was stirred for 2 h. The obtained precipitates were centrifuged and washed with methanol for several times and dried in vacuum at 60 °C to obtain Fe, Co doped ZIF-8. Finally, Fe, Co doped ZIF-8 was placed in a tube furnace and heated to 1000 °C for 1 h in a stream of Ar to yield CoNP@FeNC-0.05. CoNP@FeNC-0.02 and CoNP@FeNC-0.08 were also prepared by the above method except the addition of 0.02 mmol Co(NO_3_)_2_·6H_2_O and 0.08 mmol Co(NO_3_)_2_·6H_2_O, respectively.

#### Preparation of NC

ZIF-8 was placed in a tube furnace and heated to 1000 °C for 1 h in a stream of Ar to yield NC.

### Materials Characterizations

The morphologies and structures of the prepared catalysts were studied by field emission scanning electron microscope (FESEM, JSM-7800F), transmission electron microscope (TEM, JEM-2800F), high-resolution TEM (HRTEM, JEM-2800F), aberration-corrected high-angle annular dark-field scanning transmission electron microscope (AC HAADF-STEM, JEM-ARM200F), X-ray Diffraction (XRD, Rigaku XtalAB PRO MM007 DW), and Raman spectroscopy (HORIBA LabRAM HR Evolution). N_2_ adsorption/desorption isotherms were obtained by using a Micromeritics ASAP 2460 Surface Area and Porosity Analyzer. X-ray photoelectron spectroscopy (XPS, Thermo Scientific ESCALAB 250Xi) was employed to investigate the surface composition, and chemical states of samples. Inductively coupled plasma optical emission spectrometer (ICP-OES, Agilent 725ES) was employed to investigate the accurate element contents of samples.

### Electrochemical Tests

Electrochemical tests were conducted on a CHI760E electrochemical working station with a standard three-electrode system. For the preparation of catalyst inks, 5 mg catalyst, 500 μL of distilled water, 450 μL of ethanol, and 50 μL of 5 wt.% Nafion solution were mixed and sonicated for 1 h. Then 6 μL of ink was dropped into a commercial glassy carbon (GC) electrode (AFE5TOSOGC, 5 mm of diameter, 0.196 cm^2^, Pine Research Instrumentation) and was kept at room temperature until complete drying. Carbon rods and Ag/AgCl electrodes were used as the counter and reference electrodes, respectively. N_2_/O_2_-saturated KOH was used as electrolytes. All electrochemical tests were performed at room temperature. Cyclic voltammetry (CV) was performed at a scan rate of 50 mV s^−1^, and linear sweep voltammetry (LSV) curves were recorded at a scan rate of 5 mV s^−1^ with a rotating speed of 1600 rpm. Tafel plots were derived from relevant LSV curves. All potentials in this work were normalized to a reversible hydrogen electrode (RHE) according to the Nernst equation (*E*_RHE_ = *E*_Ag/AgCl_ + 0.197 V + 0.059 pH). Rotating ring-disk electrode (RRDE) voltammograms were recorded to obtain the peroxide yield (${\mathrm{HO}}_{2}^{-} \left(\mathrm{\%}\right)$ and number of transferred electrons (*n*) according to the equations below:

1 $$ \mathrm{HO}_{2}^{-} \quad (\%) \; = \; \frac{2I_{\text{d}}/N}{I_{\text{d}}+I_{\text{r}}/N}$$

2 $$n = \frac{{4I_{{\text{d}}} }}{{I_{{\text{d}}} + I_{{\text{r}}} /N}}$$

where *I*_d_ is disk current, *I*_r_ is ring current, and *N* is current collection efficiency of the Pt ring and is determined to be 0.41.

### Zn-air Batteries

Home-made rechargeable Zn-air batteries were assembled with stainless-steel meshes (current collectors), polished zinc plates (anodes), electrolytes, separators, carbon clothes coated with active materials (cathodes), and gas diffusion layers in turn. The loading of catalysts on carbon clothes was 2.5 mg cm^−2^. The separators (Celgard 2340) have thickness of 38 μm, porosity of 45%, pore size of 0.035 μm and good hydrophilicity. The gas diffusion layer (PLM0) was purchased from Changsha Spring New Energy Company. The electrolytes contained 6 M KOH and 0.2 M Zn(Ac)_2_. Before testing, the assembled Zn-air batteries were kept static for 1 h.

## Results and Discussion

### Materials Characterization

**Fig. 1:**
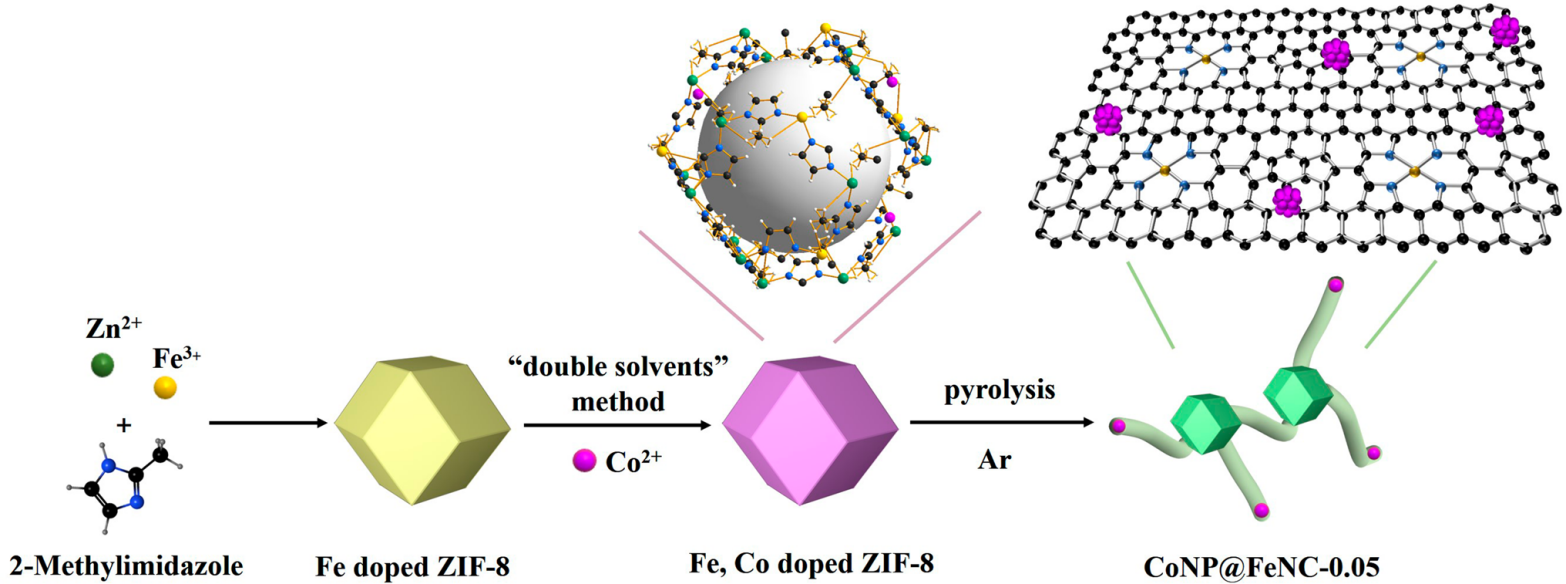
Schematic illustration for the fabrication of CoNP@FeNC

The preparation method of CoNP@FeNC-0.05 is shown in Fig. 1. First, Fe and Zn ions as metal nodes connected with 2-methylimidazole to form Fe-doped ZIF-8 [40], which was beneficial for the uniform doping of Fe. Then Fe-doped ZIF-8 was dispersed in n-hexane, and methanol containing cobaltous nitrate was dropwise added into the above dispersion. Taking advantage of the “double solvents” method, Co^2+^ was mainly incorporated into the cavities of Fe-doped ZIF-8 rather than adsorbed on the surface of Fe-doped ZIF-8 polyhedrons due to the difference of hydrophilism between the two solvents [41, 42], which were helpful for the diameter reduction and uniform distribution of produced Co nanoparticles. According to the XRD patterns (Fig. S1), Fe, Co doped ZIF-8 has similar crystal structure to that of ZIF-8. Finally, CoNP@FeNC-0.05 formed by pyrolysis of Fe, Co doped ZIF-8 at 1000 °C under Ar. During the pyrolysis process, Zn nodes were reduced and volatilized (Zinc, boiling point = 907 °C), which could promote the formation of porous structures [35]. For comparison, Fe-doped ZIF-8 and Fe, Co doped ZIF-8 were also calcined at 1000 °C under Ar (denoted as FeNC and CoNP@NC, respectively) (Figs. S2 and S3).

**Fig. 2:**
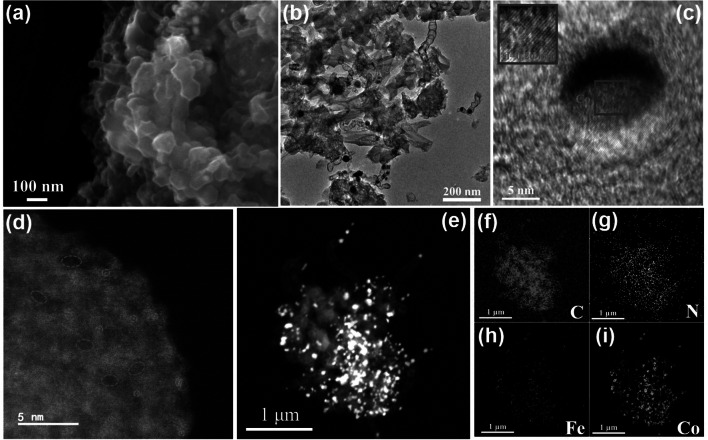
**a** SEM images of CoNP@FeNC-0.05. **b** TEM and **c** HRTEM images of CoNP@FeNC-0.05. **d** AC HAADF-STEM image of CoNP@FeNC-0.05 (orange circles: highly dispersed Fe; green circle: pores). **e–i** TEM image and corresponding EDS mapping of CoNP@FeNC-0.05

The morphologies of the prepared catalysts were disclosed by SEM and TEM. As revealed by SEM images (Fig. 2a), hollow carbon polyhedrons and carbon nanotubes (CNTs) both exist in CoNP@FeNC-0.05. The diameters of CNTs are ~ 30 nm. Moreover, the TEM image (Fig. 2b) indicates that hollow carbon polyhedrons and CNTs interconnect to form three-dimensional (3D) carbon networks and Co nanoparticles are mainly encapsulated in CNTs with an average diameter of ~ 25 nm. The presence of metallic Co nanoparticles in CoNP@FeNC-0.05 was evidenced by HRTEM (Fig. 2c). The distance between adjacent lattice fringes is determined to be 0.20 nm, corresponding to metallic Co(111) planes. Meanwhile, from the selected area electron diffraction (SAED) of CoNP@FeNC-0.05 (Fig. S4), Co nanoparticles have a polycrystalline structure. From AC HAADF-STEM image (Fig. 2d), the isolated bright spots distributed on carbon substrates, further indicate the high dispersion of Fe species within the CoNP@FeNC-0.05 catalyst. Abundant pores are also observed. Energy dispersive spectroscopy (EDS) mapping provides the element distribution of CoNP@FeNC-0.05 (Fig. 2e–i). Fe and N elements are uniformly distributed on the whole carbon substrate, while Co atoms gather as nanoparticles. Moreover, the contents of metal elements were determined by ICP-OES (Table S1). CoNP@FeNC-0.05 have a close Fe content (1.04 wt.%) with FeNC (1.05 wt.%), and have more Co species (4.02 wt.%) than CoNP@NC (1.82 wt.%) probably due to the anchoring function of dispersed Fe sites for Co species [43]. The element contents of catalysts were also determined by XPS (Table S2). The Fe contents of CoNP@FeNC-0.05 determined from ICP-OES and XPS are consistent, while Co content of CoNP@FeNC-0.05 from XPS is lower than that from ICP-OES since the Co nanoparticle are coated by CNTs.

**Fig. 3:**
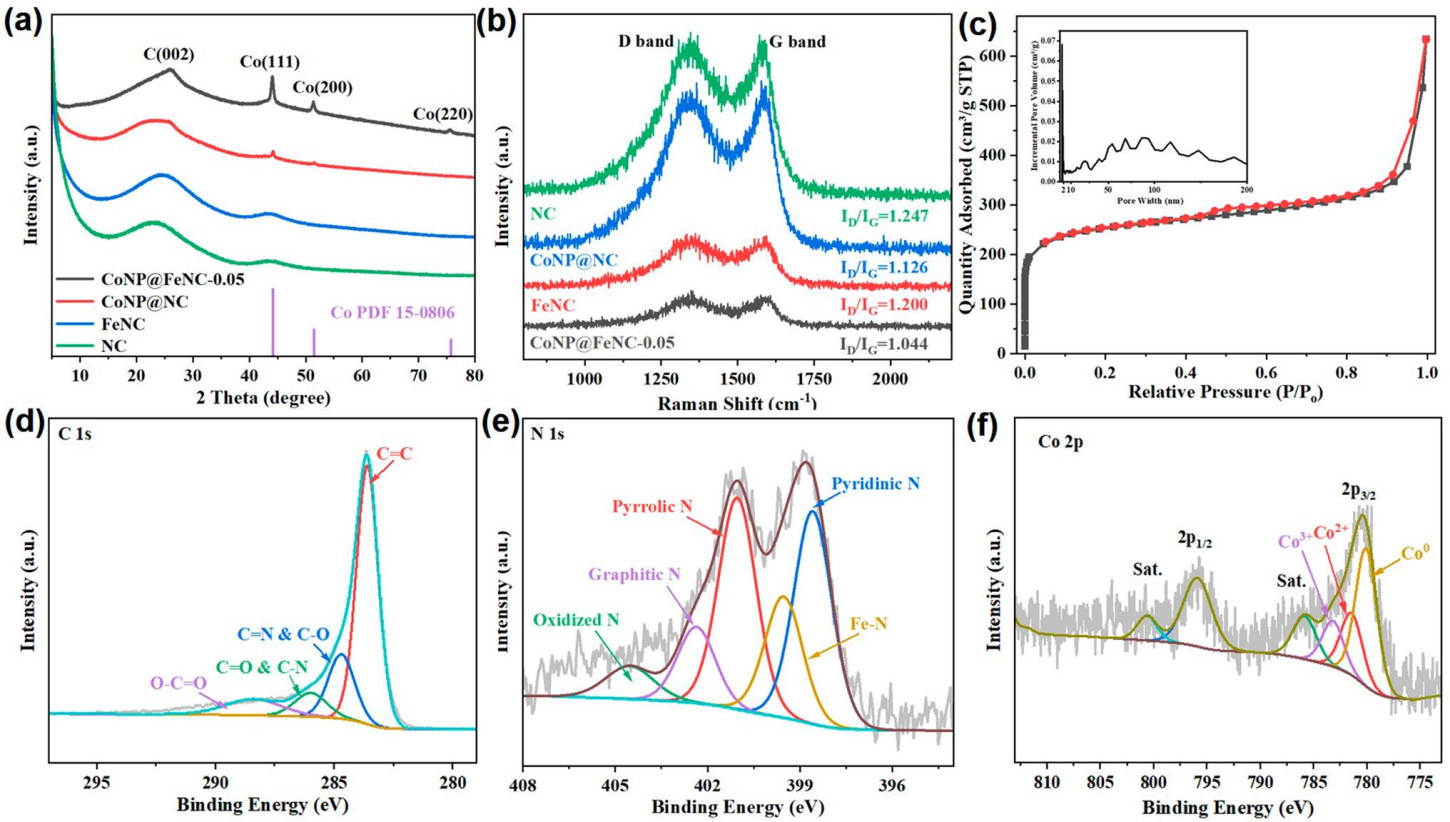
**a** XRD patterns of CoNP@FeNC-0.05, FeNC, CoNP@NC and NC. **b** Raman spectra of prepared catalysts. **c** N_2_ adsorption-desorption isotherm curve and pore size distribution of CoNP@FeNC-0.05. **d** High-resolution C 1*s* XPS of CoNP@FeNC-0.05. **e** High-resolution N 1*s* XPS of CoNP@FeNC-0.05. **f** High-resolution Co 2*p* XPS of CoNP@FeNC-0.05

The compositions and crystal structures of catalysts were investigated by XRD. For XRD patterns of CoNP@FeNC-0.05 and CoNP@NC (Fig. 3a), the peaks at 44.2°, 51.5° and 75.9° correspond to the (111), (200) and (220) crystal planes of metallic Co, respectively, indicating the presence of metallic Co nanoparticles in CoNP@FeNC-0.05 and CoNP@NC. Meanwhile, for CoNP@FeNC-0.05, CoNP@NC, FeNC and NC (Fig. 3a), the broad peaks at ~ 24° and ~ 43° are attributed to the diffraction of the (002) and (101) planes of graphitic carbons, respectively. Note that no obvious peaks associated with Fe-based crystal structures appear for XRD patterns of CoNP@FeNC-0.05 and FeNC, probably due to the low content (1.04 wt.%) and high dispersion of Fe in catalysts. Moreover, Raman spectra of catalysts were collected to explore the graphitic degrees of carbon substrates (Fig. 3b). The bands located at ~ 1340 and ~ 1588 cm^−1^, correspond to *D* band (defective/disordered *sp*^3^ hybridized carbon) and *G* band (the crystallized graphitic *sp*^2^ carbon), respectively [7]. Therefore, the intensity ratio of *D* band to *G* band (*I*_*D*_/*I*_*G*_) could reflect the graphitization degrees of prepared catalysts. The *I*_*D*_/*I*_*G*_ values of CoNP@NC and FeNC are higher than that of NC and are lower than that of CoNP@FeNC-0.05, indicating that the incorporation of metal could improve the graphitization degree of carbon substrates [44]. The high graphitization degrees of catalysts are beneficial for electron transport. The surface areas and pore distributions of catalysts could be determined by N_2_ adsorption-desorption tests. The N_2_ adsorption-desorption isotherm of CoNP@FeNC-0.05 is shown in Fig. 3c, which is close to type-IV isotherm. The Brunauer-Emmett-Teller (BET) surface area and pore volume of CoNP@FeNC-0.05 is 935.3 m^2^ g^−1^ and 0.64 cm^3^ g^−1^, respectively. The large surface area of CoNP@FeNC-0.05 is beneficial for the exposure of active sites. Meanwhile, the inset in Fig. 3c reveals that both micropores and mesopores exist in CoNP@FeNC-0.05. Hierarchical porous structure could improve mass transportation for electrocatalytic reactions.

XPS was further used to disclose the surface compositions and chemical states of catalysts. The existence of C, N, Co, and O in CoNP@FeNC-0.05 was also evidenced by the XPS survey spectrum (Fig. S5). The peaks attributed to Fe is not obvious in XPS survey spectrum since the low content and high dispersion of Fe atoms. However, the high-resolution Fe 2*p* XPS of CoNP@FeNC-0.05 (Fig. S6) confirms the existence of Fe. Furthermore, the high-resolution XPS of different elements was analyzed to explore chemical states of catalysts. The high-resolution C 1*s* XPS is shown in Fig. 3d; partial C atoms connect with N atoms in CoNP@FeNC-0.05, indicating the doping of N into the carbon substrates. Meanwhile, the high-resolution N 1*s* XPS of CoNP@FeNC-0.05 (Fig. 3e) could be deconvoluted into five type of N species including pyridinic N (398.6 eV), Fe/Co–N (399.5 eV), pyrrolic N (401.0 eV), graphitic N (402.4 eV) and oxidized-N (404.5 eV) [45]. According to the result, Fe coordinate with N (denoted as Fe–N) in CoNP@FeNC-0.05. By the deconvolution of Fe 2*p*_3/2_ peak in the high-resolution Fe 2*p* XPS of CoNP@FeNC-0.05 (Fig. S6), Fe exists in the form of Fe^2+^ (711.0 eV) and Fe^3+^ (713.9 eV) due to the coordination with N [40]. In addition, for high-resolution Co 2*p* XPS of CoNP@FeNC-0.05 (Fig. 3f), Co 2*p*_3/2_ and Co 2*p*_1/2_ locate at 780.5 and 796.0 eV, respectively. By the deconvolution of Co 2*p*_3/2_ peak [46], Co elements mainly exist in the metal form (780.2 eV), which is consistent with the results of HRTEM and XRD. Meanwhile, partial Co elements exist in the form of Co^2+^ (781.5 eV) and Co^3+^ (783.3 eV) in CoNP@FeNC-0.05, originating from the surface oxidation of metallic Co in air. The high-resolution O 1*s* XPS of CoNP@FeNC-0.05 further confirms the partial oxidation of Co nanoparticles (Fig. S7). According to the characterization of morphology and structure, uniform Co nanoparticles and highly dispersed Fe were successfully loaded on N-doped 3D carbon substrates.

### Electrochemical Performance

**Fig. 4:**
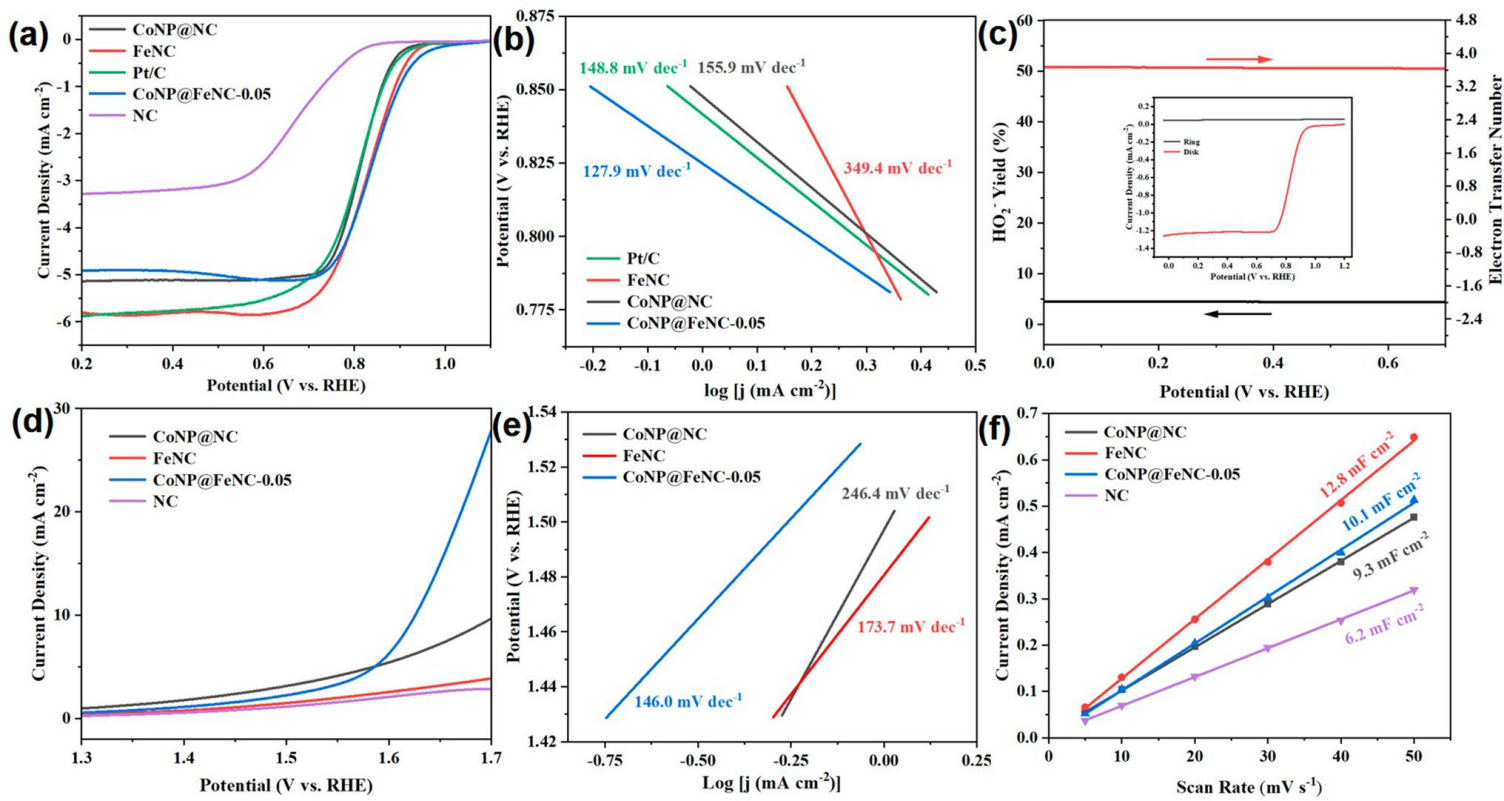
**a** LSV curves of catalysts for ORR with a scan rate of 5 mV s^−1^ under 0.1 M KOH, at 1600 rpm. **b** Tafel plots of catalysts for ORR. **c** Electron transfer number and HO_2_^−^ yield of CoNP@FeNC-0.05 derived from the RRDE tests under 0.1 M KOH. **d** LSV curves of catalysts for OER with a scan rate of 5 mV s.^−1^ under 1 M KOH, at 1600 rpm. **e** Tafel plots of catalysts for OER. **f** Plots of current density versus scan rate under 0.1 M KOH. The slopes of the curves correspond to the *C*_dl_ values of catalysts

From the above analysis, CoNP@FeNC with active transition metal elements, distinct 3D carbon networks, large surface area and porous structures, are potential ORR/OER catalysts. Therefore, the electrocatalytic performances of CoNP@FeNC materials and control catalysts were evaluated by using a standard three-electrode system in alkaline media at room temperature. Ag/AgCl electrodes, carbon rods and glass carbon coated with catalyst inks, served as the reference, counter and working electrodes, respectively. For ORR, all tests were conducted in 0.1 M KOH. As shown in Fig. S8, compared with CV curves of CoNP@FeNC-0.05, FeNC, CoNP@NC and NC under N_2_-saturated 0.1 M KOH, the CV curves of these catalysts under O_2_-saturated 0.1 M KOH all show obvious oxygen reduction peaks, indicating that these catalysts are active for ORR. Moreover, CoNP@FeNC-0.05 shows more positive half-wave potential (0.85 V vs. RHE) than the commercial Pt/C catalyst (0.82 V vs. RHE), FeNC (0.83 V vs. RHE), CoNP@NC (0.82 V vs. RHE) and NC (0.68 V vs. RHE) according to the LSV curves (Fig. 4a), which suggests that CoNP@FeNC-0.05 delivers the best activity for ORR among these catalysts. Meanwhile, the results also indicate that Co nanoparticles and highly dispersed Fe species are more active for ORR than defective carbon substrates in transition metal/carbon composite catalysts. Furthermore, by comparing the performances of CoNP@FeNC-0.05, FeNC and CoNP@NC, we found that Fe–N sites are major active centers for ORR, and the incorporation of Co nanoparticles further improve the ORR performance. SCN^−^ could strongly bind to isolated transition metal sites, and put a setback for the adsorption of O_2_ [47]. The ORR performances of CoNP@FeNC-0.05 and FeNC obviously declined after adding SCN^−^ in KOH (Fig. S9), further confirming that isolated Fe–N sites play an important role to improve ORR, as the isolated Fe–N sites may improve the adsorption of intermediates. In addition, the ORR onset potential of CoNP@FeNC-0.05 is more positive than those of other control catalysts (Table S3). The ORR performance of CoNP@FeNC-0.05 was also compared with reported advanced catalysts (Table S4), further confirming that CoNP@FeNC-0.05 is an outstanding oxygen reduction electrocatalyst. Then, the ORR kinetics of the catalysts was studied with Tafel slopes determined by *i*-*t* method (Figs. 4b and S10). As shown in Fig. 4b, CoNP@FeNC-0.05 catalyst exhibits the smallest Tafel slope among these catalysts, suggesting that CoNP@FeNC-0.05 catalyst has the best ORR kinetics. In addition, the electron transfer number and the yield of HO_2_^−^ of CoNP@FeNC-0.05 were determined by the rotating ring-disk electrode (RRDE) test. The electron transfer number of CoNP@FeNC-0.05 is close to 4, and the HO_2_^−^ yield of CoNP@FeNC-0.05 is below 5% (Fig. 4c), indicating that CoNP@FeNC-0.05 catalyst mainly experiences the efficient 4e^−^ path during ORR.

For OER, as shown in Fig. 4d, CoNP@FeNC-0.05 generates a current density of 10 mA cm^−2^ at the overpotential of 0.40 V, which is smaller than those of CoNP@NC (0.47 V), FeNC and NC, suggesting that Co nanoparticles show better activity for OER than Fe–N species. Moreover, the synergy between Co nanoparticles and Fe–N greatly enhances the OER performance of CoNP@FeNC-0.05. Meanwhile, the overpotential of CoNP@FeNC-0.05 (400 mV) at a current density of 10 mA cm^−2^ is similar to that of the commercial RuO_2_ (350 mV) (Fig. S11), confirming that CoNP@FeNC-0.05 is a promising OER catalyst. Moreover, the Tafel slope of CoNP@FeNC-0.05 (146.0 mV dec^−1^) for OER is much smaller than those of CoNP@NC (246.4 mV dec^−1^) and FeNC (173.7 mV dec^−1^) (Figs. 4e and S12), indicating that CoNP@FeNC-0.05 has faster OER kinetics than CoNP@NC and FeNC. Comparing the Tafel slopes of catalysts for ORR, we found that although Fe–N sites are active for ORR and Co nanoparticles are active for OER, individual Fe–N sites or Co nanoparticles showed poor ORR kinetics and OER kinetics, respectively. When taking advantages of the two components, CoNP@FeNC-0.05 showed excellent ORR/OER kinetics. The OER performance of CoNP@FeNC-0.05 is compared in Table S5 with reported catalysts.

In addition, the electrochemical active surface area (ECSA) was estimated by double-layer capacitance (*C*_dl_), which was obtained from the CV curves at different scan rates in the non-Faraday potential region. The *C*_dl_ of FeNC (12.8 mF cm^−2^) is higher than those of CoNP@FeNC-0.05 (10.1 mF cm^−2^), CoNP@NC (9.3 mF cm^−2^) and NC (6.2 mF cm^−2^) (Figs. 4f and S13). Although FeNC possesses larger ECSA than CoNP@FeNC-0.05, the ORR/OER performance of FeNC is worse than that of CoNP@FeNC-0.05, indicating that CoNP@FeNC-0.05 has higher intrinsic ORR/OER catalytic activity due to the synergistic effect between Fe–N and Co nanoparticles. To further investigate the synergy, the ORR and OER performances were tested for the catalyst (CoNP@NC+FeNC) of CoNP@NC mixed with FeNC. Either ORR or OER, CoNP@FeNC-0.05 exhibits superior performances than CoNP@NC+FeNC (Fig. S14). Moreover, the influences of the ratio of Fe–N to Co nanoparticles in CoNP@FeNC catalysts were investigated on ORR/OER performance. CoNP@FeNC catalysts with different ratios were prepared by the same method except the difference of the adding amount of Co(NO_3_)_2_·6H_2_O. When the adding amount of Co(NO_3_)_2_·6H_2_O is 0.05 mmol, the prepared CoNP@FeNC-0.05 catalyst showed better ORR and OER performance than FeCo_0.02_-NC and FeCo_0.08_-NC (Figs. S15 and S16).

The stability is also an important metric to evaluate the performance of electrocatalysts, in addition to the activity. For ORR, CoNP@FeNC-0.05 was subjected to the CV tests with a scan rate of 100 mV s^−1^ for 5000 cycles. The half-wave potential of CoNP@FeNC-0.05 decreases only 0.04 V by comparing the LSV curves measured before and after the durability test (Fig. S17a). For the OER stability tests, CoNP@FeNC-0.05 was loaded on carbon paper as the working electrode, and the potential of 1.67 V (vs. RHE) was applied to the electrode in 1 M KOH by *i*-*t* method. After continuous reaction of 10,000 s, the current density of CoNP@FeNC-0.05 retains 62% of the initial one (Fig. S18a). The catalysts after ORR and OER durability tests both remain the primary morphology and still possess uniform element distribution (Figs. S17 and S18), further confirming the good stability of CoNP@FeNC-0.05 under operation conditions.

According to performance tests and the characterizations of morphology and structure, the activity origin of CoNP@FeNC-0.05 for ORR/OER was summarized as follows: (I) Fe–N sites are active for ORR. Moreover, the adjacent Co nanoparticles could interact with Fe–N sites, thus improving the performance of ORR [48]. (II) Co nanoparticles contribute to the improvement of OER performance. Meanwhile, the wrapping of CNTs for Co nanoparticles could prevent the aggregation and detachment of Co nanoparticles, which are beneficial for the stability of CoNP@FeNC-0.05 under electrochemical reactions [49]. In addition, the introduction of Fe–N sites has an obvious impact on the resulting OER performance of Co-based catalysts [50, 51]. (III) Large specific surface area of CoNP@FeNC-0.05 derived from the MOF precursor promotes the exposure of active sites. Porous structures and distinct 3D carbon networks of CoNP@FeNC-0.05 are beneficial for the mass transportation and electron transfer.

### Battery Performance

**Fig. 5:**
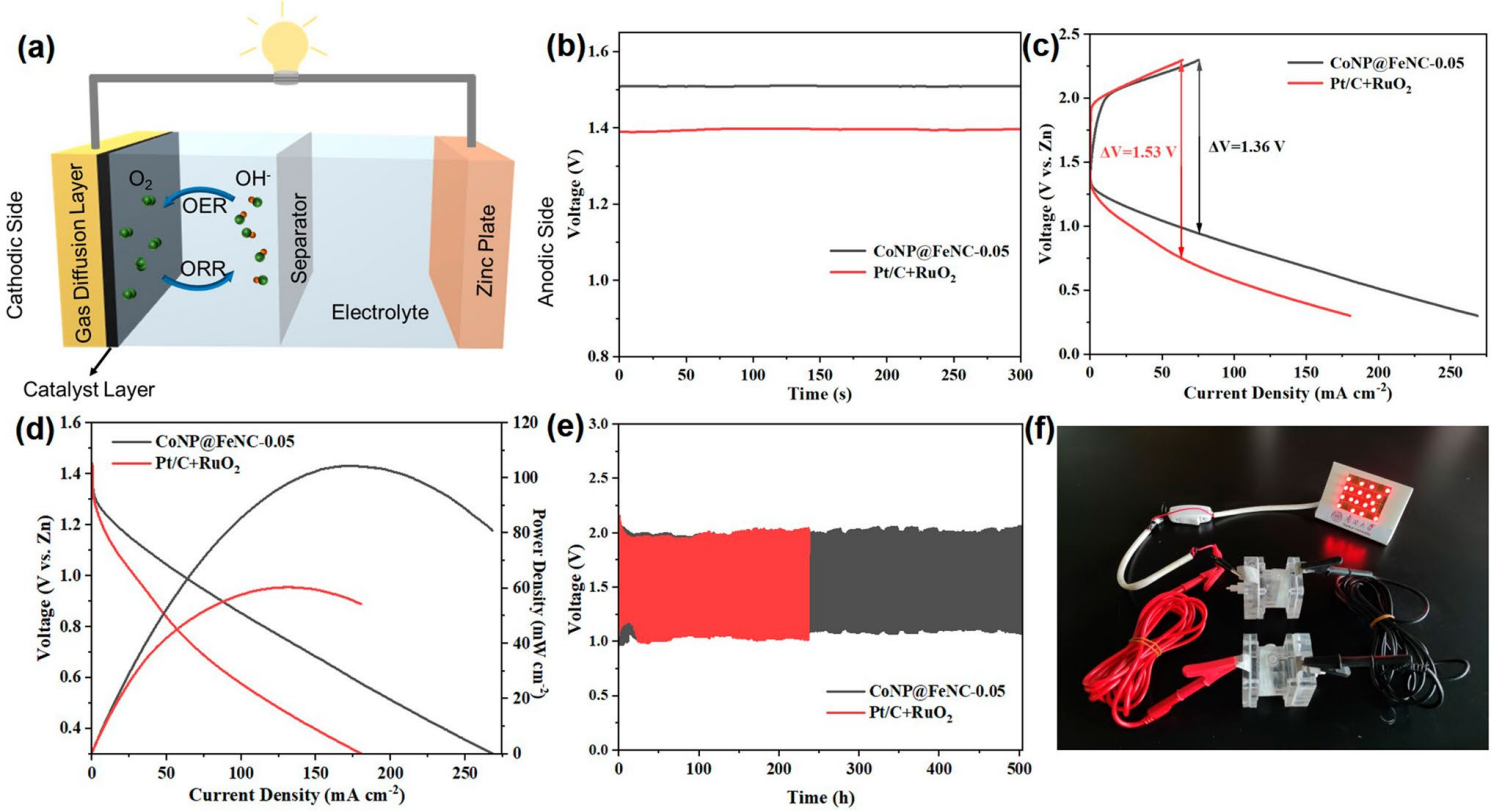
**a** Schematic diagram of rechargeable Zn-air batteries. **b** Open-circuit plots of rechargeable Zn-air batteries. **c** Charge and discharge polarization curves. **d** Discharge polarization curves and corresponding power density curves. **e** Galvanostatic discharge-charge cycling curves. **f** Photo of a lit LED powered by two Zn-air batteries of CoNP@FeNC-0.05 as cathode catalysts in series

CoNP@FeNC-0.05 is a promising candidate for ORR and OER. Thus, home-made Zn-air batteries were assembled with CoNP@FeNC-0.05 as cathode catalysts to further investigate the practical performance of CoNP@FeNC-0.05 (Fig. 5a). Commercial Pt/C and RuO_2_ catalysts were also employed as cathode catalysts of Zn-air batteries for comparison. The assembled Zn-air batteries with CoNP@FeNC-0.05 as cathode catalysts exhibit an open-circuit potential of 1.51 V, which is higher than that of Zn-air batteries with commercial catalysts as cathode catalysts (1.39 V) (Fig. 5b). Moreover, from the charge-discharge polarization curves in Fig. 5c, the voltage gap between charging and discharging processes of Zn-air batteries with CoNP@FeNC-0.05 at 75 mA cm^−2^ is 1.36 V while the voltage gap of Zn-air batteries with commercial catalysts at 63 mA cm^−2^ is 1.53 V, indicating that CoNP@FeNC-0.05 is more active for ORR/OER than Pt/C+RuO_2_. Meanwhile, according to the power density curves derived from corresponding discharge polarization curves (Fig. 5d), the peak power density of Zn-air batteries with CoNP@FeNC-0.05 as cathode catalysts is 104.4 mW cm^−2^, which is higher than that of Zn-air batteries with commercial catalysts as cathode catalysts (60.4 mW cm^−2^). When cycled at 5 mA cm^−2^ (discharging for 30 min and charging for 30 min), the Zn-air batteries with CoNP@FeNC-0.05 as cathode catalysts could stably run for 500 h, while the Zn-air batteries with commercial catalysts as cathode catalysts could stably run for 237 h (Fig. 5e). Zn-air batteries with CoNP@FeNC-0.05 as cathode catalysts demonstrate better cycling stability and rechargeability. Meanwhile, obviously the discharge voltages of Zn-air batteries with CoNP@FeNC-0.05 are higher than those of Zn-air batteries with commercial catalysts from the galvanostatic discharge–charge curves, suggesting that CoNP@FeNC-0.05 is highly active for ORR. In addition, the performance of Zn-air batteries with CoNP@FeNC-0.05 as cathode catalysts is also compared with reported results (Table S6), further confirming that CoNP@FeNC-0.05 is a promising oxygen electrocatalyst for practical applications. Moreover, for the practical application, two Zn-air batteries with CoNP@FeNC-0.05 as cathode catalysts in series could easily light up the LED light (Fig. 5f). From the above tests, CoNP@FeNC-0.05 is an extraordinary ORR/OER bifunctional catalyst for Zn-air batteries.

## Conclusions

In summary, the efficient oxygen electrocatalyst was rationally designed and successfully prepared by virtues of MOFs and two different doping methods. CoNP@FeNC-0.05 showed outstanding ORR/OER performance under an alkaline medium. When applied as cathode catalysts of rechargeable Zn-air batteries, CoNP@FeNC-0.05 played an important role in improving the performance of Zn-air batteries, particularly for peak power density and cycling stability. By performance tests and structure characterizations, the uniformly dispersed Fe–N sites and Co nanoparticles in CoNP@FeNC-0.05 contributed to the promotion of performance for ORR/OER. In addition, porous structures, large specific surface areas, and distinct 3D carbon networks of CoNP@FeNC-0.05 were also beneficial for the improvement of ORR/OER performance. This work offers a valuable reference for rational design and construction of single catalysts with diverse active centers for oxygen electrocatalysis and paves a way for the practical application of rechargeable Zn-air batteries.

## Supplementary Information

Below is the link to the electronic supplementary material.

Supplementary file1 (PDF 1840 KB)
