# Supplementary material for: MOF-Derived Co and Fe Species Loaded on N-Doped Carbon Networks as Efficient Oxygen Electrocatalysts for Zn-Air Batteries
Source: Nanomicro Lett. 2022 Aug 11;14:162. doi: 10.1007/s40820-022-00890-w (PMC9372253; doi:10.1007/s40820-022-00890-w)
Supplement: Supplementary file 1 — Supplementary file1 (PDF 1840 KB) [file 40820_2022_890_MOESM1_ESM.pdf]

Supporting Information for

MOF-Derived Co and Fe Species Loaded on N-Doped Carbon Networks as Efficient Oxygen Electrocatalysts for Zn-Air Batteries

Yuanyuan Xue[1], Yibo Guo[1], Qinming Zhang[1], Zhaojun Xie[1,*], Jinping Wei[1], and Zhen Zhou[2,*]

[1] School of Materials Science and Engineering, Institute of New Energy Material Chemistry, Key Laboratory of Advanced Energy Materials Chemistry (Ministry of Education), Renewable Energy Conversion and Storage Center (ReCast), Nankai University, Tianjin 300350, People's Republic of China

[2] School of Chemical Engineering, Zhengzhou University, Zhengzhou 450001, People's Republic of China

*Corresponding authors. E-mail: zjxie@nankai.edu.cn (Zhaojun Xie), zhenzhou@zzu.edu.cn (Zhen Zhou)

Supplementary Figures and Tables

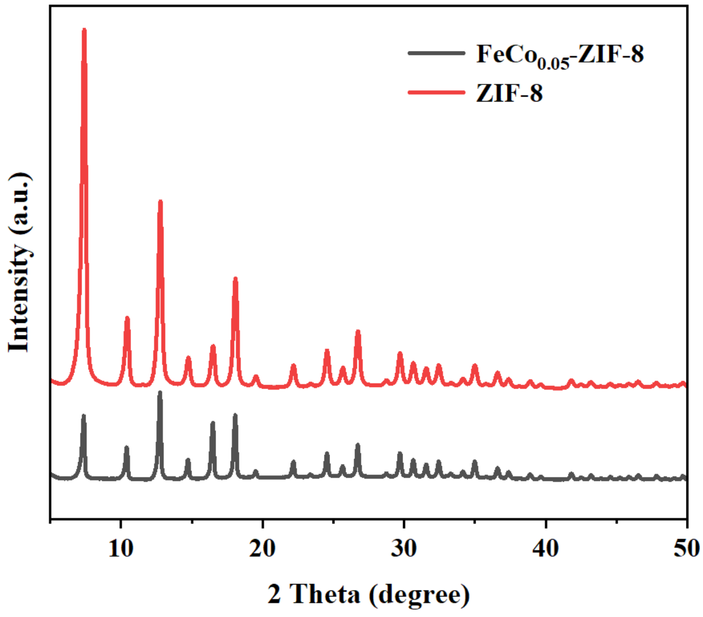

Fig. S1 XRD patterns of $FeCo_{0.05}$-ZIF-8 and ZIF-8

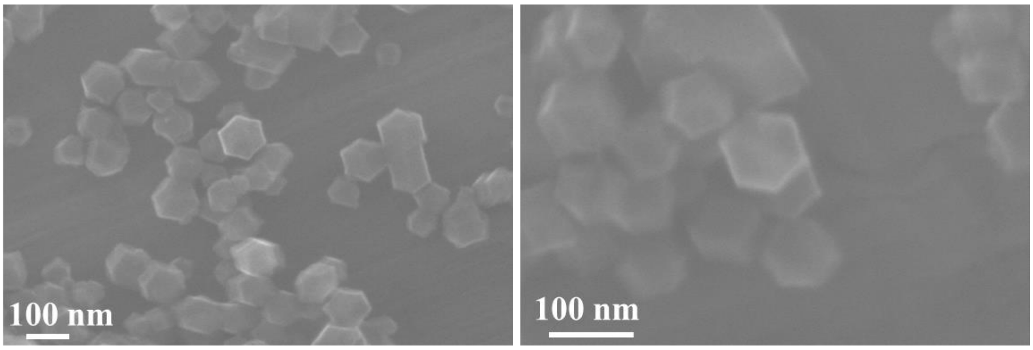

Fig. S2 SEM images of FeNC

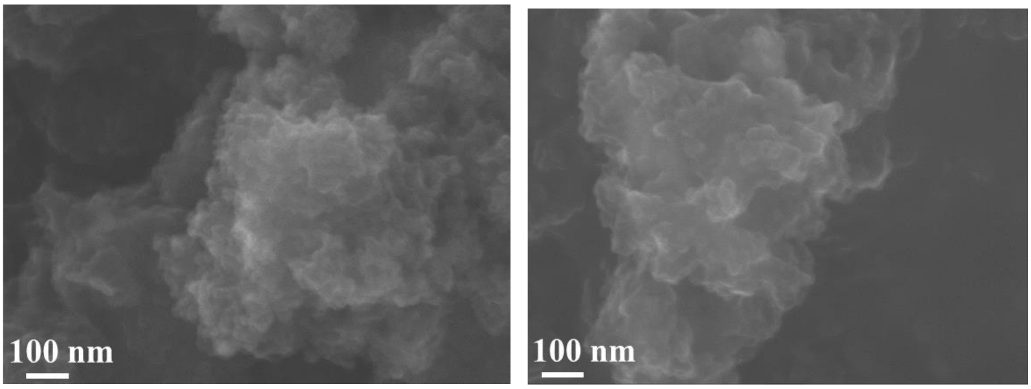

Fig. S3 SEM images of CoNP@NC

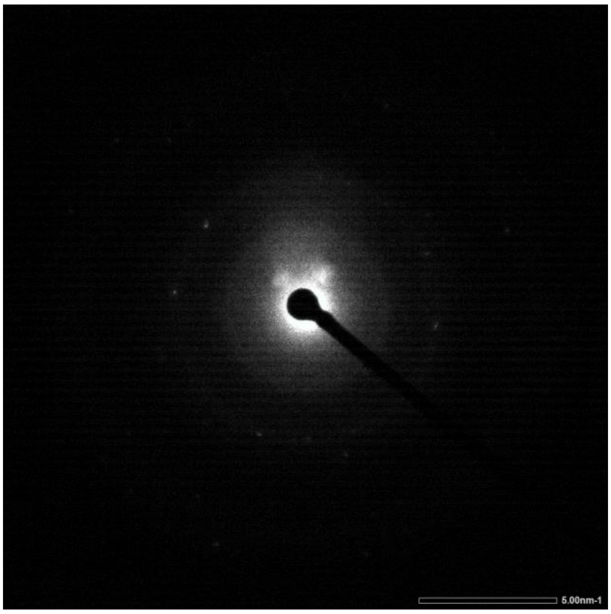

Fig. S4 SAED image of CoNP@FeNC-0.05

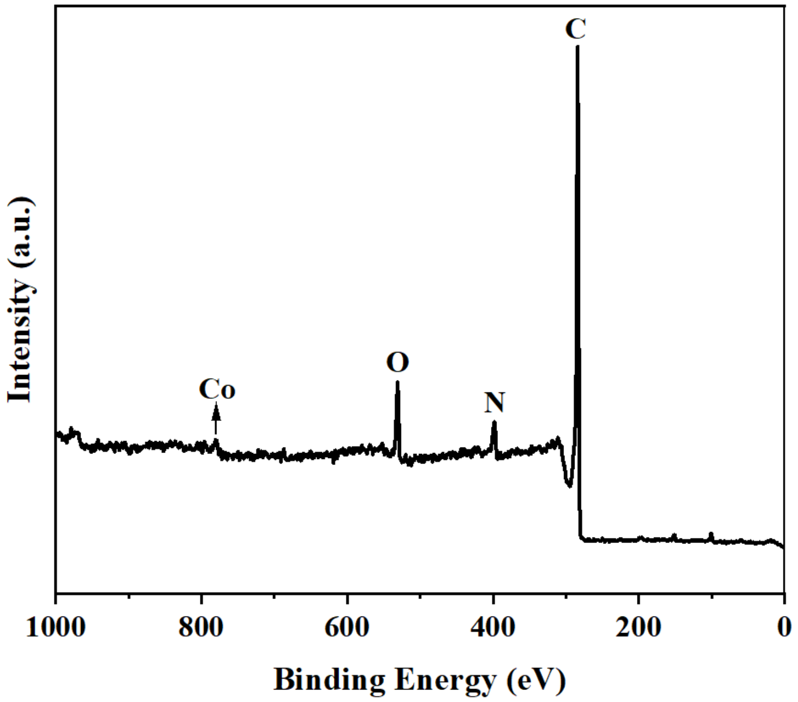

Fig. S5 XPS survey spectrum of CoNP@FeNC-0.05

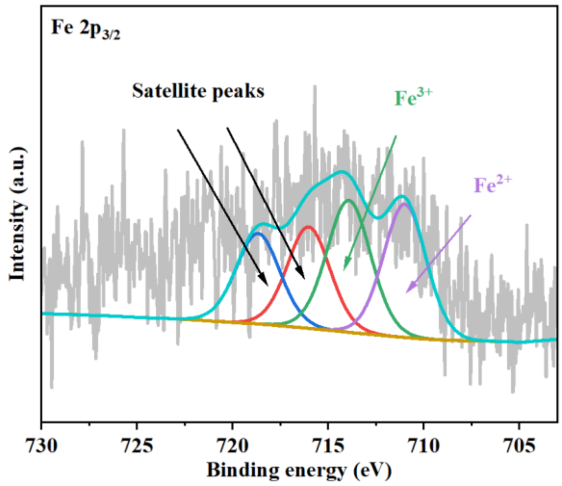

Fig. S6 High-resolution Fe 2p XPS of CoNP@FeNC-0.05

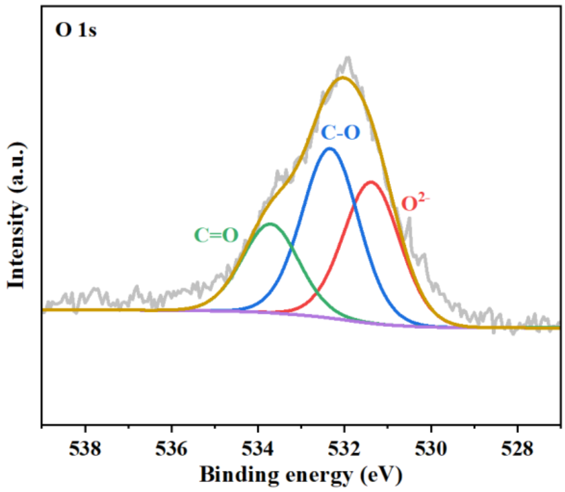

Fig. S7 High-resolution O 1s XPS of CoNP@FeNC-0.05

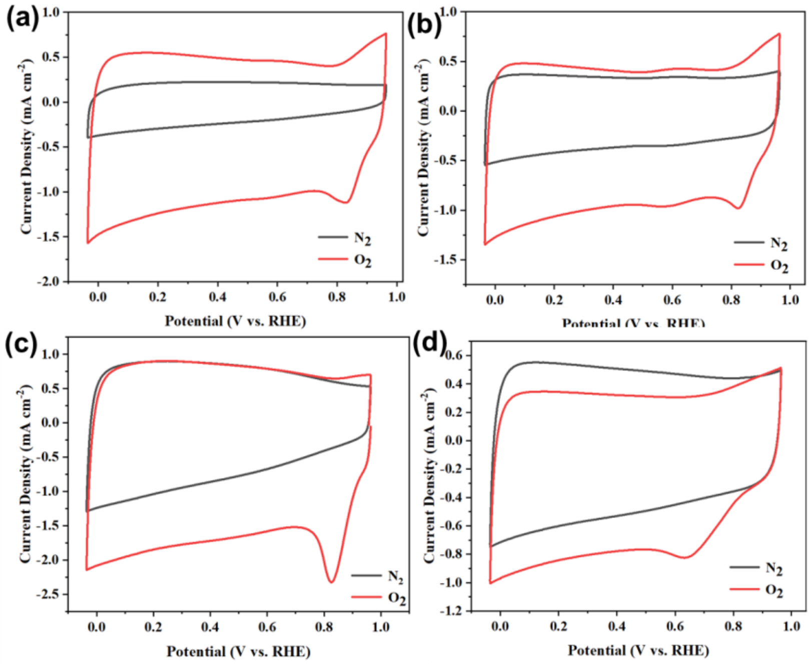

Fig. S8 CV curves of (**a**) CoNP@FeNC-0.05, (**b**) CoNP@NC, (**c**) FeNC, (**d**) NC under N_2 or O_2 saturated 0.1 M KOH for ORR

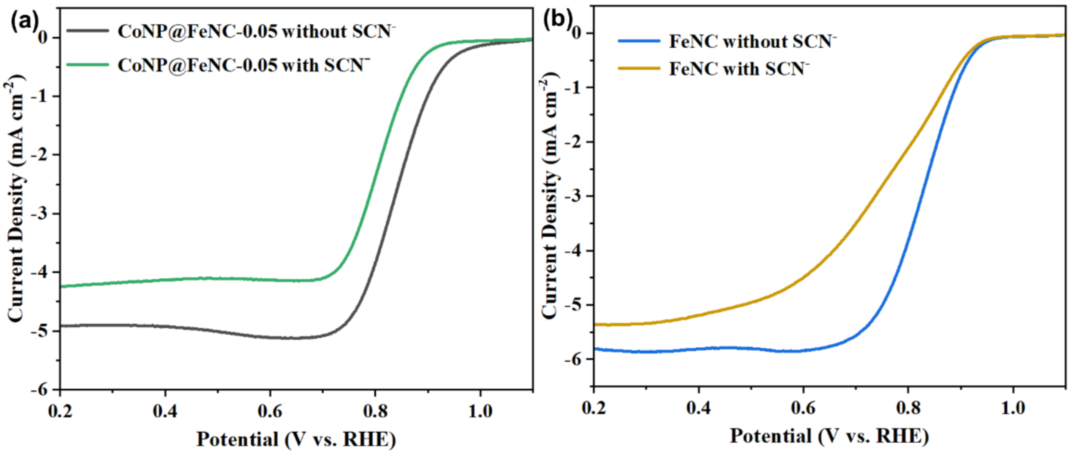

Fig. S9 LSV curves of catalysts for OER under 0.1 M KOH, measured before and after adding SCN^- (0.1 M)

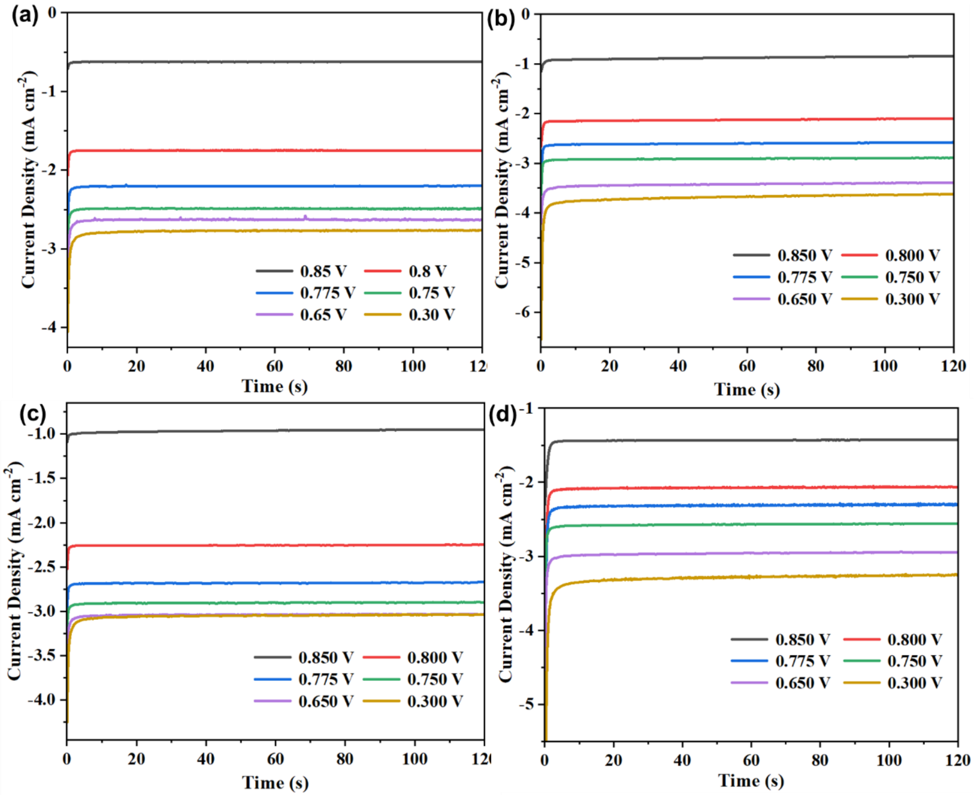

Fig. S10 The i-t curves of (**a**) CoNP@FeNC-0.05, (**b**) Pt/C, (**c**) CoNP@NC, and (**d**) FeNC, under 0.1 M KOH, with a rotation speed of 800 rpm

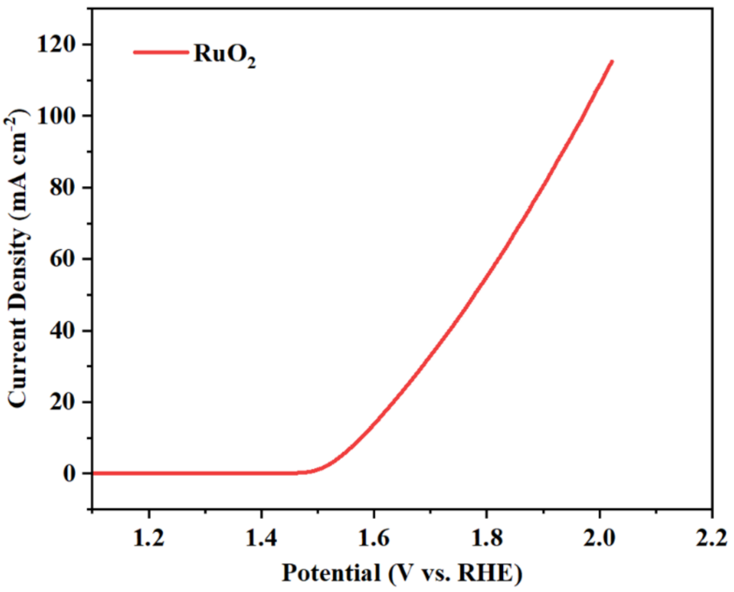

Fig. S11 LSV curve of RuO_2 catalyst for OER with a scan rate of 5 mV s^{-1} under 1 M KOH, at 1600 rpm

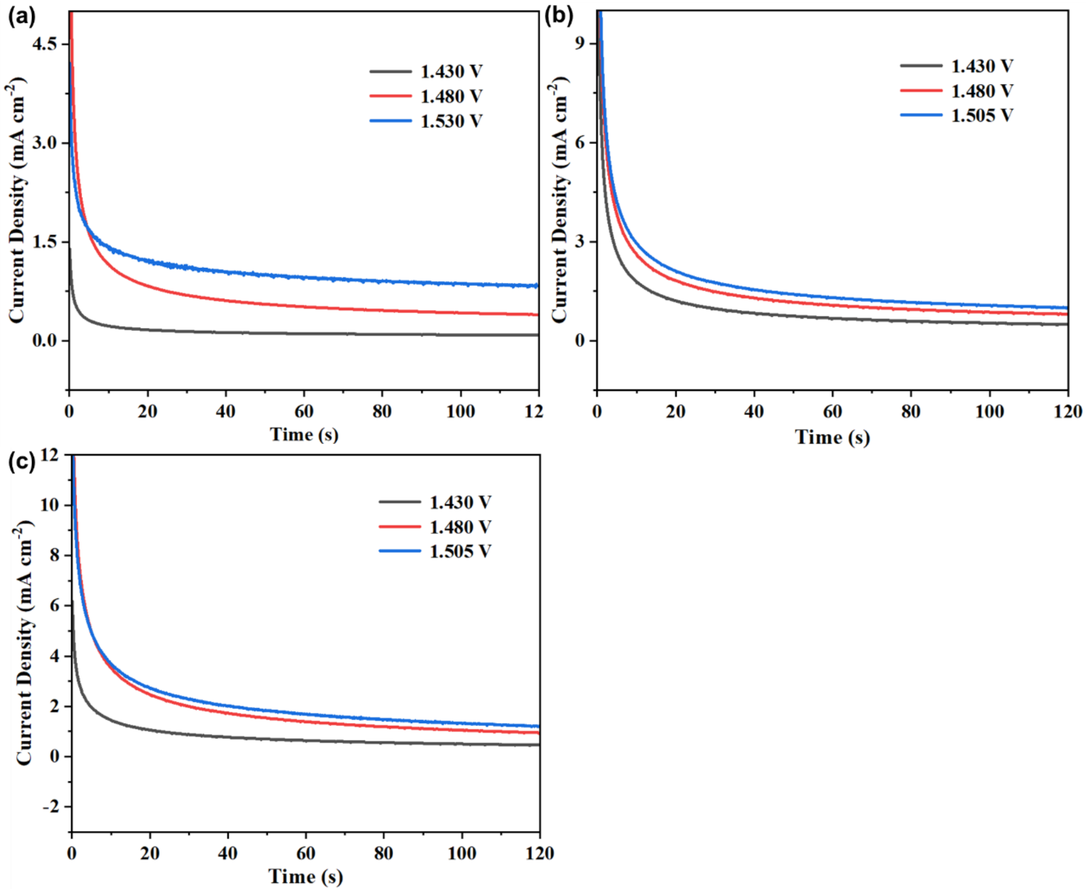

Fig. S12 The i-t curves of (**a**) CoNP@FeNC-0.05, (**b**) CoNP@NC, and (**c**) FeNC, under 1 M KOH, with a rotation speed of 800 rpm

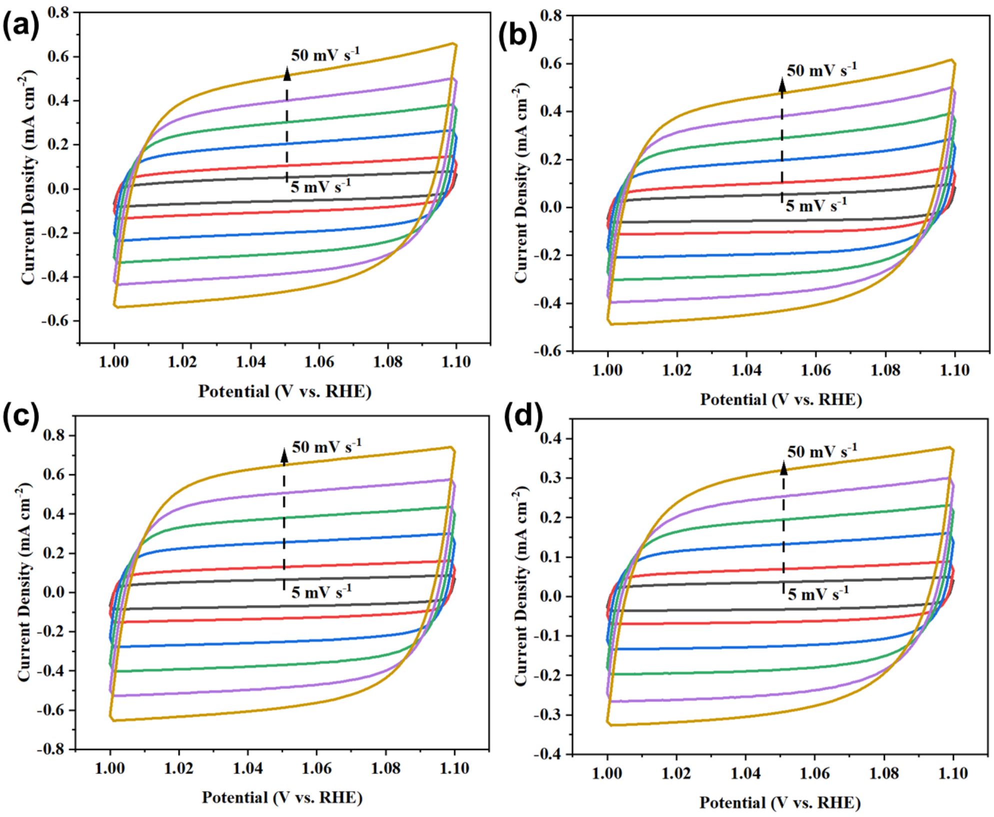

Fig. S13 CV curves at various scan rates within a potential window from 1.0 to 1.1 V vs. RHE without Faradaic processes: (**a**) CoNP@FeNC-0.05, (**b**) CoNP@NC, (**c**) FeNC, and (**d**) NC

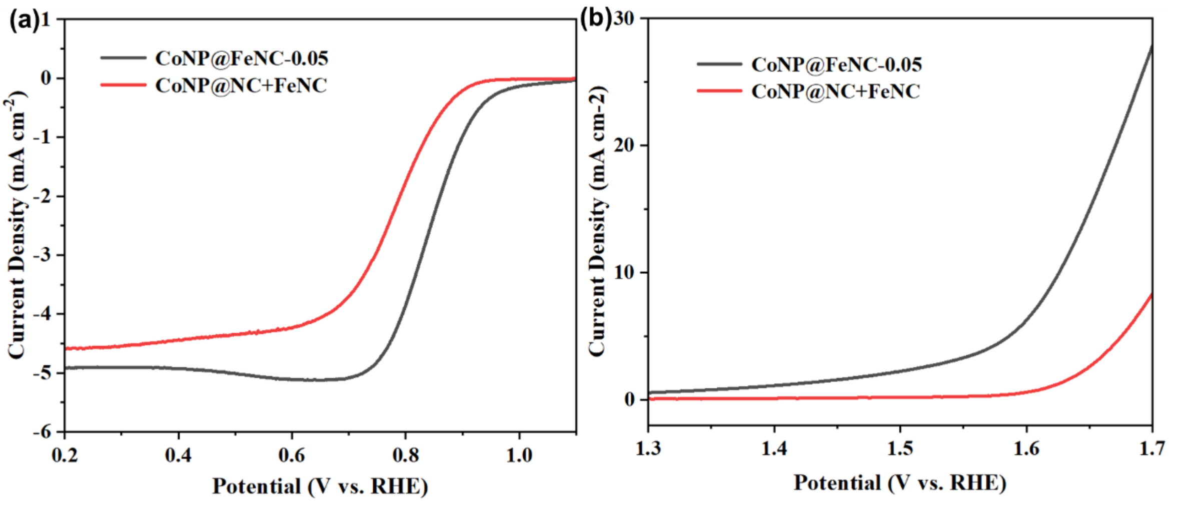

Fig. S14 (**a**) LSV curves of CoNP@FeNC-0.05 and CoNP@NC+FeNC for ORR under 0.1 M KOH. (**b**) LSV curves of CoNP@FeNC-0.05 and CoNP@NC+FeNC for OER under 1 M KOH

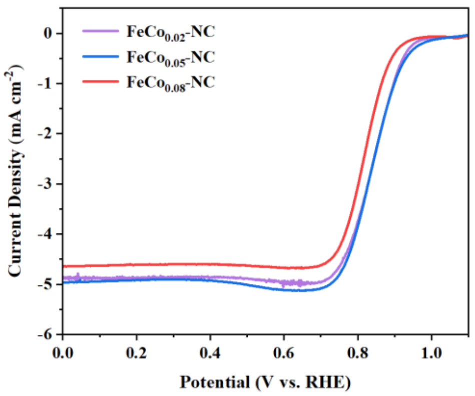

Fig. S15 LSV curves of CoNP@FeNC catalysts for ORR with a scan rate of 5 mV s^{-1} under 0.1 M KOH, at 1600 rpm

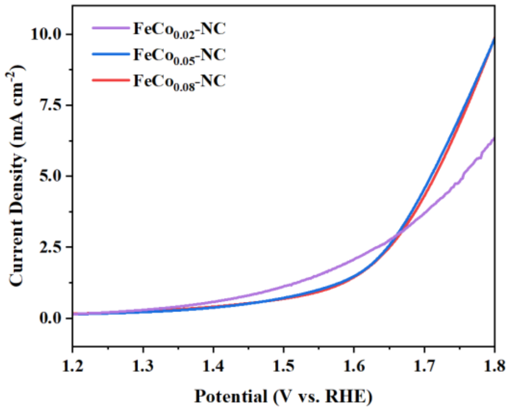

Fig. S16 LSV curves of CoNP@FeNC catalysts for OER with a scan rate of 5 mV s^{-1} under 1 M KOH, at 1600 rpm

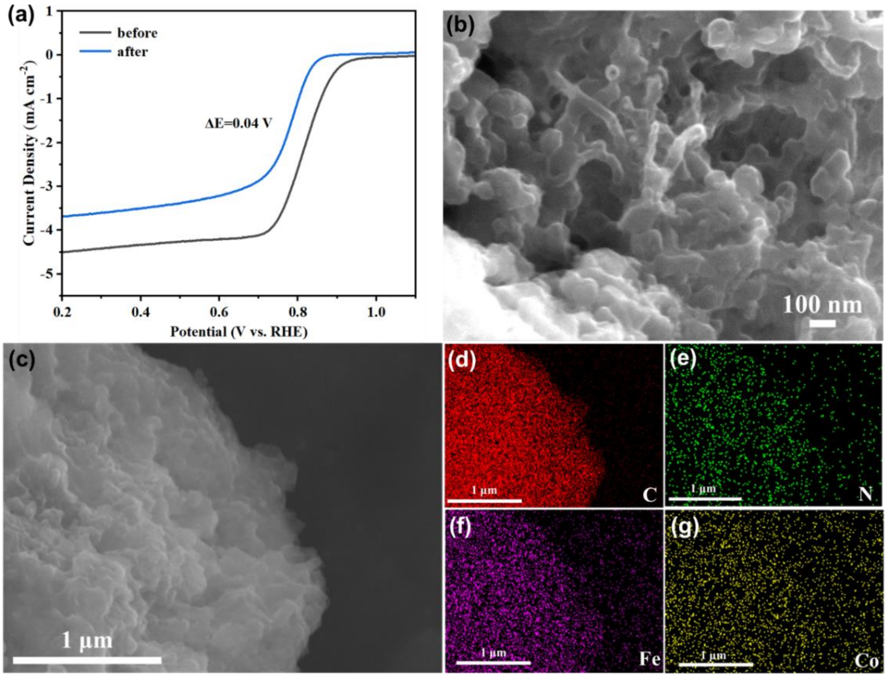

Fig. S17 (**a**) LSV curves of CoNP@FeNC-0.05 for ORR, before and after CV tests of 5000 cycles under 0.1 M KOH. (**b**) SEM image of CoNP@FeNC-0.05 after CV tests of 5000 cycles. (**c-g**) SEM image and corresponding EDS mapping of CoNP@FeNC-0.05 after CV tests of 5000 cycles

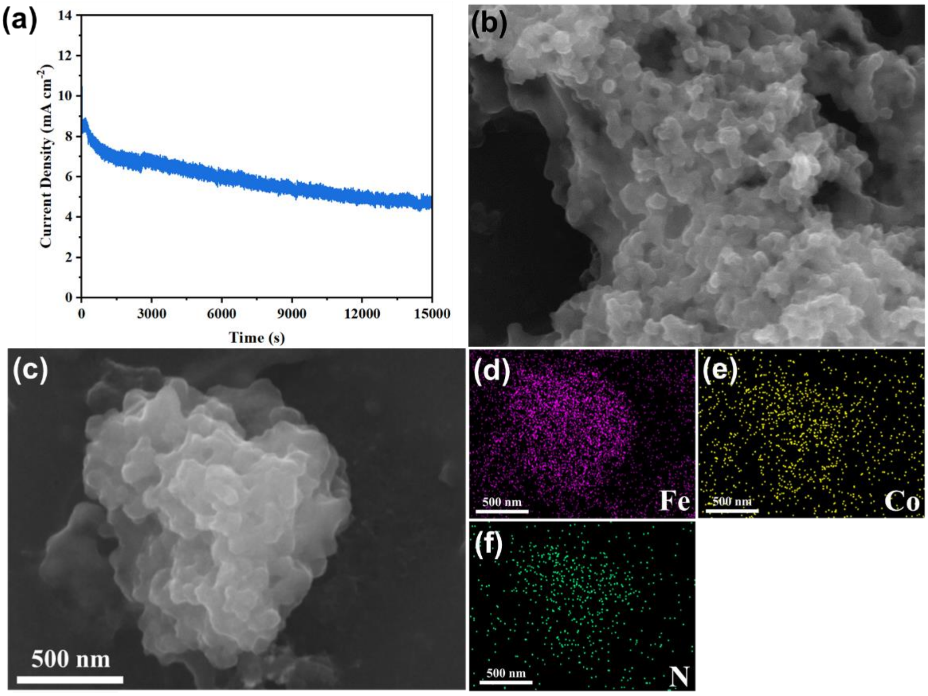

Fig. S18 (**a**) The i-t curve of CoNP@FeNC-0.05 for OER at 1.67 V under 1 M KOH. (**b**) SEM image of CoNP@FeNC-0.05 after 15000-s reaction. (**c-f**) SEM image and corresponding EDS mapping of CoNP@FeNC-0.05 after 15000-s reaction. The distribution of carbon is not offered since CoNP@FeNC-0.05 was loaded on carbon paper under durability tests

Table S1 The element contents of prepared catalysts from ICP-OES tests

	**FeNC**	**CoNP@NC**	**CoNP@FeNC-0.05**
**Fe (wt%)**	1.05	-	1.04
**Co (wt%)**	-	1.82	4.02

Table S2 The element contents of prepared catalysts from XPS tests

	**FeNC**	**CoNP@NC**	**CoNP@FeNC-0.05**
**Fe (wt%)**	1.46	-	1.41
**Co (wt%)**	-	2.45	1.87
**C (wt%)**	83.33	85.11	82.00
**O (wt%)**	8.08	6.82	8.38
**N (wt%)**	7.13	5.62	6.33

Table S3 Comparisons of ORR performance (vs. RHE) of CoNP@FeNC-0.05 with control catalysts in 0.1 M KOH

	NC	CoNP@NC	FeNC	CoNP@FeNC-0.05	Pt/C
**Half-wave potential (V)**	0.68	0.82	0.83	0.85	0.82
**Onset potential (at -0.1 mA cm^{-2}) (V)**	0.85	0.95	0.96	1.02	0.98

Table S4 Comparison of ORR performance (vs. RHE) of CoNP@FeNC-0.05 with reported related catalysts in 0.1 M KO

Catalysts	Half-wave potential (V)	Onset potential (V)	Scan rate (mV s^{-1})	Rotation sate (rpm)	Refs.
CoNP@FeNC-0.05	0.85	1.02	5	1600	**This work**
FeCo-N-HCN	0.86	0.98	-	1600	Adv. Funct. Mater. 2021, 31, 2011289.
Co–NHC-900	0.85	-	10	1600	ACS Appl. Mater. Interfaces 2017, 9, 38499-38506.
PPy/FeTCPP/Co	0.86	1.01	10	1600	Adv. Funct. Mater. 2017, 27, 1606497.
Fe,Co,N-CNP(0.3)	0.875	0.979	-	1600	ACS Appl. Mater. Interfaces 2018, 10, 12651-12658.
Fe_3C-Co/NC	0.885	-	10	1600	Adv. Funct. Mater. 2019, 29, 1901949.
COF@ZIF_{800}	0.85	0.99	-	1600	J. Mater. Chem. A 2022, 10, 228-233.
Co@NPC/C-MWCNTs	0.79	0.87	10	1600	Chem. Eng. J. 2022, 432, 134192.
CoNC-CNF-1000	0.8	-	10	1600	Small 2018, 14, 1800423.
Fe/Meso-NC-1000	0.885	0.97	5	1600	Adv. Mater. 2022, 34, 2107291.
Fe/N/S-PCNT	0.84	0.96	10	1600	J. Mater. Chem. A 2019, 7, 1607-1615.
Co-SAs@NC	0.82	0.96	5	1600	Angew. Chem. Int. Ed. 2019, 58, 5359-5364.
Co-N_3C_1@GC	0.846	0.904	10	1600	ACS Catal. 2020, 10, 5862-5870.
Fe_1-HNC-500-850	0.842	0.93	5	-	Adv. Mater. 2020, 32, 1906905.

Table S5 Comparison of OER performance of CoNP@FeNC-0.05 with reported related catalysts (vs. RHE)

Catalysts	Potential at j=10 mA cm^{-2} (V)	Electrolyte	Scan rate (mV s^{-1})	Rotation rate (rpm)	Refs.
CoNP@FeNC-0.05	1.63	1 M KOH	5	1600	**This work**
Fe_3C-Co/NC	1.57	1 M KOH	5	-	Adv. Funct. Mater. 2019, 29, 1901949.
CF–NG–Co	1.63	1 M KOH	5	1600	J. Mater. Chem. A 2018, 6, 489-497.
PPy/FeTCPP/Co	1.61	0.1 M KOH	10	1600	Adv. Funct. Mater. 2017, 27, 1606497.
CoO@Co/N-rGO	1.65	0.1 M KOH	5	1600	J. Mater. Chem. A 2017, 5, 5865-5872.
Co/N–C-800	1.504	1 M KOH	10	-	ACS Appl. Mater. Interfaces 2022, 14, 8549-8556.
CoN-HPCNF-900	1.63	0.1 M KOH	5	-	Chem. Eng. J. 2021, 407, 127157.
Co/NGC-3	1.626	0.1 M KOH	5	1600	ACS Appl. Mater. Interfaces 2020, 12, 5717-5729.
CoNC-CNF-1000	1.68	0.1 M KOH	10	1600	Small 2018, 14, 1800423.
CoFe/Co@NCNT/NG	1.611	0.1 M KOH	10	1600	J. Power Sources 2020, 449, 227512.
Co@NHCC-800	1.512	1 M KOH	10	-	Appl. Catal. B 2019, 254, 55-65.
Co/N-CNTs (1500 sccm)	1.54	1 M KOH	5	1600	Small 2020, 16, 2002427.
Co@CNT-NC	1.633	0.1 M KOH	10	-	J. Power Sources 2022, 527, 231205.

Table S6 Comparison of performance of Zn-air batteries of CoNP@FeNC-0.05 with reported related results.

Catalysts	Open-circuit potential (V)	Maximum Power density (mW cm^{-2})	Cycling stability	Refs.
CoNP@FeNC-0.05	1.51	104.4	500 cycles (60 min/cycle) @5 mA cm^{-2}	**This work**
CoNi-SAs/NC	1.45	101.4	95 cycles (20 min/cycle) @5 mA cm^{-2}	Adv. Mater. 2019, 31, 1905622.
FeCo-DACs/NC	1.50	175	480 cycles (30 min/cycle) @10 mA cm^{-2}	Adv. Mater. 2022, 34, 2107421.
(Fe,Co)-SA/CS	1.43	86.65	300 cycles (20 min/cycle) @5 mA cm^{-2}	Small Methods 2021, 5, 2000751.
Fe/Co-N/S-Cs	1.395	102.63	80 cycles (20 min/cycle) @5 mA cm^{-2}	Appl. Catal. B 2019, 241, 95-103.
Fe-N4 SAs/NPC	-	232	108 cycles (20 min/cycle) @2 mA cm^{-2}	Angew. Chem. Int. Ed. 2018, 57, 8614-8618.
FeNx/C-700-20	1.6	36	-	Adv. Energy Mater. 2018, 8, 1800955.
SAFe-SWCNT film	1.47	210	-	Appl. Catal. B 2021, 294, 120239.

Fe/N-G-SAC	-	120	240 cycles (60 min/cycle) @10 mA cm^{-2}	Adv. Mater. 2020, 32, 2004900.
CoNC-NB2	1.50	246	420 cycles (20 min/cycle) @2 mA cm^{-2}	Small 2020, 16, 2001171.
CNT@SAC-Co/NCP	1.45	172	100 cycles (20 min/cycle) @5 mA cm^{-2}	Adv. Funct. Mater. 2021, 31, 2103360.
H-Co@FeCo/N/C	1.45	125.2	400 cycles (30 min/cycle) @10 mA cm^{-2}	Appl. Catal. B 2020, 278, 119259.
Co@hNCTs-800	1.45	149	3000 cycles (10 min/cycle) @5 mA cm^{-2}	Nano Energy 2020, 71, 104592.
Co@Co_3O_4@NC-900	0.842	64	100 cycles (120 min/cycle) @5 mA cm^{-2}	J. Mater. Chem. A 2018, 6, 1443-1453.
